# Sign-specific stimulation ‘hot’ and ‘cold’ spots in Parkinson’s disease validated with machine learning

**DOI:** 10.1093/braincomms/fcab027

**Published:** 2021-03-10

**Authors:** Alexandre Boutet, Jurgen Germann, Dave Gwun, Aaron Loh, Gavin J B Elias, Clemens Neudorfer, Michelle Paff, Andreas Horn, Andrea A Kuhn, Renato P Munhoz, Suneil K Kalia, Mojgan Hodaie, Walter Kucharczyk, Alfonso Fasano, Andres M Lozano

**Affiliations:** 1 Joint Department of Medical Imaging, University of Toronto, Toronto, ON, Canada; 2 University Health Network, Toronto, ON, Canada; 3 Department of Neurology, Charité – Universitätsmedizin Berlin, Freie Universität Berlin, Humboldt-Universität zu Berlin, and Berlin Institute of Health, Germany; 4 Berlin School of Mind and Brain, Humboldt-Universität zu Berlin, Germany; 5 Deutsches Zentrum für Neurodegenerative Erkrankungen, Berlin, Germany; 6 Neurocure Cluster of Excellence, Charité – Universitätsmedizin Berlin, Berlin, Germany; 7 Edmond J. Safra Program in Parkinson’s Disease, Morton and Gloria Shulman Movement Disorders Clinic, Toronto Western Hospital, UHN, Division of Neurology, University of Toronto, Toronto, ON, Canada; 8 Department of Neurosurgery, University of Toronto, Toronto, ON, Canada; 9 Center for Advancing Neurotechnological Innovation to Application (CRANIA), Toronto, ON, Canada; 10 Krembil Brain Institute, Toronto, ON, Canada

**Keywords:** deep brain stimulation, Parkinson’s disease, subthalamic nucleus, neuroimaging, machine learning

## Abstract

Deep brain stimulation of the subthalamic nucleus has become a standard therapy for Parkinson’s disease. Despite extensive experience, however, the precise target of optimal stimulation and the relationship between site of stimulation and alleviation of individual signs remains unclear. We examined whether machine learning could predict the benefits in specific Parkinsonian signs when informed by precise locations of stimulation. We studied 275 Parkinson’s disease patients who underwent subthalamic nucleus deep brain stimulation between 2003 and 2018. We selected pre-deep brain stimulation and best available post-deep brain stimulation scores from motor items of the Unified Parkinson's Disease Rating Scale (UPDRS-III) to discern sign-specific changes attributable to deep brain stimulation. Volumes of tissue activated were computed and weighted by (i) tremor, (ii) rigidity, (iii) bradykinesia and (iv) axial signs changes. Then, sign-specific sites of optimal (‘hot spots’) and suboptimal efficacy (‘cold spots’) were defined. These areas were subsequently validated using machine learning prediction of sign-specific outcomes with in-sample and out-of-sample data (*n* = 51 subthalamic nucleus deep brain stimulation patients from another institution). Tremor and rigidity hot spots were largely located outside and dorsolateral to the subthalamic nucleus whereas hot spots for bradykinesia and axial signs had larger overlap with the subthalamic nucleus. Using volume of tissue activated overlap with sign-specific hot and cold spots, support vector machine classified patients into quartiles of efficacy with ≥92% accuracy. The accuracy remained high (68–98%) when only considering volume of tissue activated overlap with hot spots but was markedly lower (41–72%) when only using cold spots. The model also performed poorly (44–48%) when using only stimulation voltage, irrespective of stimulation location. Out-of-sample validation accuracy was ≥96% when using volume of tissue activated overlap with the sign-specific hot and cold spots. In two independent datasets, distinct brain areas could predict sign-specific clinical changes in Parkinson’s disease patients with subthalamic nucleus deep brain stimulation. With future prospective validation, these findings could individualize stimulation delivery to optimize quality of life improvement.

## Teaser

Boutet and Germann et al. examined whether machine learning could predict clinical benefits in specific Parkinsonian signs when informed by the precise location of stimulation. They report that machine learning modelling using distinct brain areas could predict sign-specific clinical changes in Parkinson’s disease patients with subthalamic nucleus deep brain stimulation.

## Introduction

A wide range of brain disorders is thought to arise from aberrant brain circuits.^1^ Deep brain stimulation (DBS) is a neurosurgical treatment directed towards modulating dysfunctional circuits. DBS is most established in the treatment of Parkinson’s disease.^2^ The therapeutic effects achieved with DBS surgery hinge upon the precise and selective modulation of the intended target structure, maximizing treatment efficacy while minimizing off-target spill-over into neighbouring structures to avoid adverse effects.^3^ In Parkinson’s disease, the most commonly targeted brain structure is the subthalamic nucleus (STN), an essential hub in the brain’s motor circuitry.^1^ Despite extensive experience, however, questions persist about the optimal stimulation target for Parkinson’s disease, and more specifically, the precise characterization of the neural substrates responsible for sign-specific improvements.^4^

Parkinson’s disease patients typically express cardinal motor signs of tremor, rigidity, bradykinesia and axial signs to varying degrees.^5^ Functional neuroimaging studies have suggested that clinical features map to different brain networks, forming the basis for sign-specific *circuitopathies.*^1^ In STN-DBS for Parkinson’s disease, structural connections between STN and the supplementary motor area were associated with bradykinesia and rigidity improvement, while structural connections with the primary motor cortex were linked to tremor alleviation.^6^ Symptom-specific networks have also been implicated in psychiatric disorders such as obsessive-compulsive disorder, in which obsessions/compulsions mapped to different white matter bundles than depressive symptoms.^7^ These findings suggest that appropriately delivered network modulation could lead to feature-specific, rather than disease-specific effects, potentially allowing for individualized DBS therapy based on patients’ unique clinical profile.

Previous studies have investigated the relationship between clinical feature-specific improvement and stimulation location in STN-DBS. While global motor improvement seems to consistently localize to the dorsolateral STN region,^8^ sign-specific sites of optimal stimulation differ across studies. This may partly reflect the heterogenous methods employed in previous studies, as well as limited statistical power. For example, a study^8^ found an area encompassing dorsolateral STN and neighbouring white matter was associated with rigidity improvement, whereas another group^6^ defined a rigidity improvement zone that was limited to the dorsolateral STN. The latter also defined a tremor improvement zone in dorsolateral STN more antero-lateral than that of rigidity. In line with these concepts, targets nearby to STN, such as the dentato-rubro thalamic tract, have been prospectively investigated to preferentially treat tremor-dominant Parkinson’s disease.^9^

The primary purpose of this study was not necessarily to investigate anatomical structures responsible for clinical benefits, but rather to define the relationship between improvement of Parkinson’s disease cardinal signs and stimulation location in a large sample of patients treated with STN-DBS. To do so, we modelled activation volumes and employed voxel-wise linear regression to define sign-specific discriminative sites of optimal and suboptimal efficacy. We then used machine learning to determine whether sign-specific improvement could be explained by the recruitment of these discrete areas, and validated the resulting model using out-of-sample data from another institution.

## Materials and methods

### Patient population

Following institutional research ethics board approval (University Health Network Research Ethics Board #15–9777), we retrospectively screened the charts of Parkinson’s disease patients who underwent DBS surgery at Toronto Western Hospital (TWH) from 2003 to 2018. In total, 275 Parkinson’s disease patients who underwent bilateral STN-DBS at TWH were included (Table 1). Inclusion and exclusion criteria are detailed in the Supplementary material. These patients were reported in a prior study that described the relationship between volume of tissue activated (VTA) location and global motor improvement.^10^ The current study expands on this by investigating Parkinson’s disease sign-specific changes and characterizing their relationships with stimulated brain areas. An additional cohort of previously published patients from Charité-Universitätsmedizin Berlin (CUB, *n* = 51) was included as an independent test-cohort. Demographic details and imaging parameters are published elsewhere.^11^^,^^12^

### Clinical scores

As detailed in a previous study,^10^ preoperative baseline (Med-OFF) and postoperative (Med-OFF/DBS-ON) motor item scores on the Unified Parkinson's Disease Rating Scale (UPDRS-III) were collected for each patient as a measure of clinical improvement attributable to DBS. Each patient’s best available clinical postoperative score was sampled, and the corresponding stimulation parameters were recorded (Supplementary Table 1). Patients underwent extensive programming according to our published algorithms.^13^^,^^14^ Then, we selected items from UPDRS-III to calculate sign-specific change. We reviewed the literature for previous publications using clinical feature-specific neuroimaging analysis to extract the following items: (i) items 20 and 21 (rest and action tremor) for tremor, (ii) items 22 (rigidity) for rigidity, (iii) items 18, 19, 23–26 (speech, facial expression, finger tap, hand movement, rapid alternating movements of hands and leg agility) for bradykinesia and (iv) items 27–30 (arising from chair, posture, gait and axial signs) for axial signs.^8^^,^^15–17^

**Figure 1:**
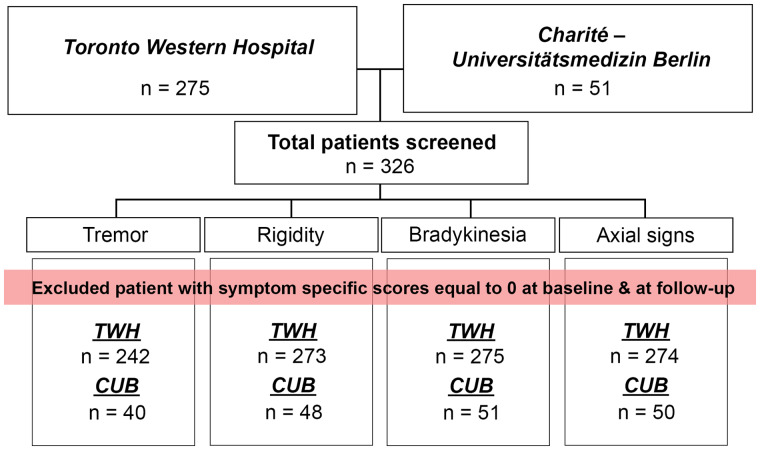
**Patient flowchart.** Two patient cohorts were included in this study (*n* = 326): TWH (*n* = 275) and CUB (*n* = 51). Because no clinical change attributable to DBS could be calculated, patients with both baseline and best postoperative scores of 0 for a specific sign were not included in the sign-specific analysis. Therefore, the number of patients included in each sign-specific analysis for TWH was: (i) tremor (*n* = 242), (ii) rigidity (*n* = 273), (iii) bradykinesia (*n* = 275) and (iv) axial signs (*n* = 274). Similarly, the number of patients included in each sign-specific analysis for CUB was: (i) tremor (*n* = 40), (ii) rigidity (*n* = 48), (iii) bradykinesia (*n* = 51) and (iv) axial signs (*n* = 50). CUB = Charité-Universitätsmedizin Berlin; TWH = Toronto Western Hospital.

The clinical change attributable to DBS was calculated based on the absolute difference between the best available postoperative score (at any time point longer than 2 months after surgery) and the baseline score. A positive difference indicated improvement whereas a negative difference indicated worsening. Absolute difference rather than per cent change was used because the maximum possible scores for each sign cluster (tremor = 28, rigidity = 20, bradykinesia = 40, axial signs = 16)—and thus the maximum change—were relatively small compared to the global UPDRS-III. As such, using percentage improvement would have resulted in large changes for patients with mild signs at baseline. Patients with both baseline and best postoperative scores of 0 for a given sign were not included in that sign-specific analysis, as no clinical change attributable to DBS could be calculated. Therefore, the number of patients included in each sign-specific analysis was: (i) tremor (*n* = 242), (ii) rigidity (*n* = 273), (iii) bradykinesia (*n* = 275) and (iv) axial signs (*n* = 274) (Fig. 1). Baseline demographics were not significantly different across these four sign-specific cohorts (*P* > 0.05, ANOVA). Mean active contact coordinates were also similar across the four signs (Supplementary Table 2 and Supplementary Fig. 1). Finally, to account for the natural history of Parkinson’s disease, the clinical improvement attributable to DBS was adjusted for disease severity using the corresponding preoperative baseline (Med-ON) and postoperative UPDRS-III scores (Med-ON/DBS-ON). Of note, this only led to minor changes in clinical scores (see Supplementary material and Supplementary Table 3).

### Image acquisition, electrode localization and VTA estimation

These steps, in keeping with previously published methods, are detailed in the Supplementary material and were performed using Lead-DBS v2.0 software (https://www.lead-dbs.org/ 20 July 2020, date last accessed).^11^ Briefly, DBS electrodes were localized and warped to standard space for group analysis. To approximate the spatial extent of the peri-electrode electric field wherein modulation of neuronal activity is assumed to occur, activation volumes were computed. Although not technically accurate, these will be referred as VTAs for context and ease of reading, especially for readers familiar with the prior clinical literature Finally, to facilitate group-level analysis, left-sided VTAs were flipped non-linearly—accounting for anatomical asymmetry—to the right using Lead-DBS.^8^^,^^11^

### Defining sign-specific sites of optimal and suboptimal efficacy

To define sign-specific sites of optimal (‘hot spots’) and suboptimal (‘cold spots’) efficacy, voxel-wise mass univariate linear regression analyses were performed using clinically weighted (by each sign cluster) VTAs. This resulted in a map of *t*-values discriminating voxels (i.e. brain areas) associated with optimal (positive *t*-values) and suboptimal (negative *t*-values) sign-specific outcomes when stimulated. Here, negative *t*-values signify that the mean sign-specific clinical change associated with overlapping VTAs compared unfavourably to that of non-overlapping VTAs, thus representing suboptimal benefits rather than worsening *per se*.

To define hot and cold spots, the sign-specific *t*-value maps obtained from the mass univariate analyses were thresholded at *P*_uncorrected_ < 0.01 and subsequently binarized. This resulted in a total of eight binary labels: Hot and cold spots for tremor (Hot_T_ and Cold_T_), rigidity (Hot_R_ and Cold_R_), bradykinesia (Hot_B_ and Cold_B_) and axial signs (Hot_A_ and Cold_A_). Overlap between each label and STN, sensorimotor STN and zona incerta was also assessed.^18^

### Internal validation of sign-specific sites of optimal and suboptimal efficacy

To validate the sign-specific hot and cold spots, as well as the chosen *P*-value threshold, we used a supervised machine learning model, specifically support vector machine (SVM). SVM approaches have previously been used to classify and predict clinical outcomes following DBS for dystonia based on neuroimaging features.^19^ For our purposes, the SVM input was the volume of overlap between individual VTAs—belonging to each sign-specific cohort—and the corresponding hot and cold spots labels. Voltage alone was also used as an input to a separate SVM to investigate the influence of VTA size (independent of VTA location) on outcome. SVM output in both cases was the classification of patients into sign-specific quartiles of predicted clinical changes. The slight differences into the quartile group size were due to patients having equal clinical score change.

### External validation of sign-specific sites of optimal and suboptimal efficacy

To provide additional external validation of the hot and cold spots, we tested our SVM model on an out-of-sample Parkinson’s disease STN-DBS dataset from a separate institution (CUB) (Fig. 1). The sign-specific clinical improvement attributable to DBS was calculated in the same manner as for the TWH cohort, based on the absolute difference between available postoperative (1–2 years after surgery) and baseline scores of the same UPDRS-III items.

Once again, two SVM models were tested. The overlap of sign-specific CUB VTAs with the corresponding hot and cold spots (derived from TWH cohort) was used as input for the first model, whereas voltage alone served as input for the second. SVM output was the classification of CUB patients into sign-specific quartiles of predicted clinical changes.

### Sign-specific structural connectivity

As an additional exploratory analysis to investigate patterns of structural connectivity between sign-specific hot spots and motor regions-of-interest previously implicated with motor improvement,^6^^,^^12^^,^^20^ we seeded Hot_T_, Hot_R_, Hot_B_ and Hot_A_ in a large averaged diffusion template created from the diffusion-weighted imaging scans of 1065 Human Connectome Project (http://www.humanconnectomeproject.org/ 15 June 2020, date last accessed) subjects.^21^ Additional details on acquisition parameters and tractography processing steps are detailed in the Supplementary material.

### Analyses using different hot and cold spots

For completeness sake, we also defined hot and cold spots using two additional analyses: (i) using the complete UPDRS-III with similar thresholding (*P*_uncorrected_ < 0.01) and binarization and (ii) using threshold cluster free enhancement to compute sign-specific hot and cold spots without binarization to use the complete statistical information.

### Statistical analyses

R (https://www.r-project.org/ 15 June 2020, date last accessed, version 3.4.4) and RMINC (https://github.com/Mouse-Imaging-Centre/RMINC 20 July 2020, date last accessed) were used (Supplementary material).

### Data availability

The validated hot and cold spots labels for each sign are publicly available for download on ZENODO (https://doi.org/10.5281/zenodo.4425256 20 January 2021, date last accessed) and are accessible in Lead-DBS (https://www.lead-dbs.org/ 20 July 2020, date last accessed). The data and code that support the central findings of this study are available from the corresponding author upon reasonable request.

## Results

### Patient characteristics

Patient demographics are shown in Table 1. Baseline demographics between the two institutions were not significantly different (*P* > 0.05, two-sample *t*-test), except for levodopa equivalent dose (*P* < 0.01) (Table 1). Mean coordinates of the active contacts differed between TWH and CUB cohorts (Supplementary Table 2). In both cohorts, tremor and rigidity improved the most in response to STN-DBS, whereas bradykinesia improved the least (Table 2). For TWH patients, the best scores for rigidity, gait and bradykinesia were achieved between 1.5 and 2 years post-DBS implantation. However, achieving best tremor control took significantly longer (2.2 years, ANOVA *P* < 0.001).

**Table 1:** Patient demographics of TWH (*n* = 275) and CUB (*n* = 51)

Patient cohort	TWH	CUB	*P*
No. of patients (female)	275 (82)	51 (17)	>0.05
Age (year)	59.8 ± 7.1	60.0 ± 7.9	>0.05
Disease duration (year)	11.4 ± 4.3	10.4 ± 3.9	>0.05
Preoperative LED (mg)	1405.1 ± 698.2	1071.7 ± 528.5	**<0.01**

Baseline demographics between the two institutions (TWH and CUB) were not significantly different (*P* > 0.05, two-sample *T*-test), except for levodopa equivalent dose (*P* < 0.01). Bold indicates statistical significance. Data are numbers of participants or mean ± standard deviation.

CUB = Charité-Universitätsmedizin Berlin; LED = levodopa equivalent dose; TWH = Toronto Western Hospital.

**Table 2:** Sign-specific clinical outcomes

Sign	Preoperative UPDRS-III score	Adjusted clinical change	Time after surgery (year)
TWH			
Tremor (*n* = 242)	6.0 ± 4.3	5.0 ± 4.2 (82.2)	2.2 ± 2.0
Rigidity (*n* = 273)	6.8 ± 3.8	4.3 ± 3.7 (60.0)	1.9 ± 1.7
Bradykinesia (*n* = 275)	16.4 ± 5.3	7.4 ± 6.2 (45.1)	1.5 ± 1.5
Axial signs (*n* = 274)	5.7 ± 2.6	3.2 ± 2.8 (53.8)	1.6 ± 1.5
CUB			
Tremor (*n* = 40)	6.5 ± 5.5	4.3 ± 3.7 (72.2)	N/A*
Rigidity (*n* = 48)	7.6 ± 4.0	4.4 ± 2.8 (57.9)	N/A*
Bradykinesia (*n* = 51)	18.1 ± 7.2	7.2 ± 5.8 (39.8)	N/A*
Axial signs (*n* = 50)	5.8 ± 3.1	2.7 ± 2.2 (46.6)	N/A*

Clinical change reflects the adjusted difference between the sign-specific UPDRS-III at the time of follow-up and prior to surgery. The clinical improvement attributable to DBS was adjusted for disease severity using the corresponding preoperative baseline (Med-ON) and postoperative UPDRS-III scores (Med-ON/DBS-ON) (see Supplementary material and Supplementary Table 2). Time after surgery represents the timepoint with best clinical score. For the CUB cohort, * indicates that the precise time after surgery was not available but it was usually 1–2 years after surgery. Unless otherwise stated the data are mean ± standard deviation and percentages in parentheses.

CUB = Charité-Universitätsmedizin Berlin; LED = levodopa equivalent dose; N/A = not available; TWH = Toronto Western Hospital; UPDRS-III: Motor section of the Unified Parkinson’s disease rating scale.

### Anatomical relationship of sign-specific sites of optimal and suboptimal efficacy

**Figure 2:**
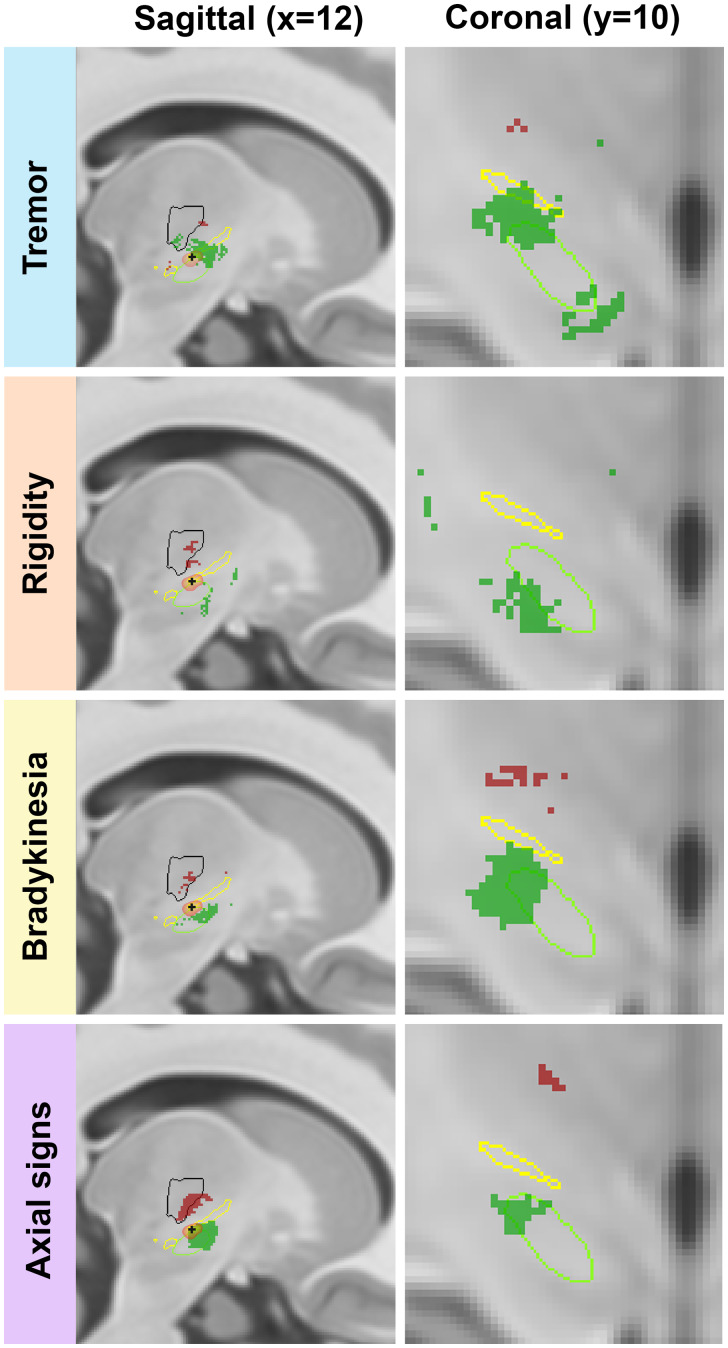
**Sign-specific area of clinical change after STN-DBS.** Binarized areas of optimal (‘hot spots’) and suboptimal (‘cold spots’) efficacy were identified using mass univariate analysis (uncorrected *P* < 0.01). Hot (positive *t*-values, green) and cold (negative *t*-values, red) spots are shown. The subthalamic nucleus (green outline), zona incerta (yellow outline), and thalamic ventral-intermediate nucleus (black outline) are projected on sagittal (first column) and coronal (second column) T_1_-weighted MRI (MNI ICBM 2009 b NLIN asymmetric). The nuclei outline were derived from nuclei labels^18^ using FSLeyes for visualization.

We used a voxel-wise mass univariate analysis (thresholded at *P*_uncorrected_ < 0.01) to define sign-specific discriminative sites of optimal (‘hot spots’) and suboptimal (‘cold spots’) efficacy. These hot and cold spots were then internally (TWH cohort) and externally (CUB cohort) validated using machine learning (SVM) classification to predict sign-specific clinical changes. The spatial distribution of hot and cold spots corresponding to each sign can be seen in Fig. 2. Both Hot_B_ and Hot_A_ primarily occupied dorsolateral STN with extension into the adjacent white matter (Fig. 2 and Supplementary Table 4). Conversely, a large portion of Hot_T_ was located outside dorsolateral STN, overlapping with zona incerta and also lying adjacent to mediodorsal STN. Most of Hot_R_ was located outside lateral STN, within the white matter located antero-ventral and antero-dorsal to STN. Cold_B_ and Cold_A_ were located in the thalamic ventral-intermediate nucleus area and adjacent white matter. Small foci of Cold_T_ straddled the inferior thalamus and adjacent white matter. Cold_R_ was located in ventral-intermediate nucleus and nearby white matter. Hot_R_ exhibited 24% and 17% overlap with Hot_B_ and Hot_A,_ respectively.

### Machine learning validations of sign-specific sites of optimal and suboptimal efficacy

**Figure 3:**
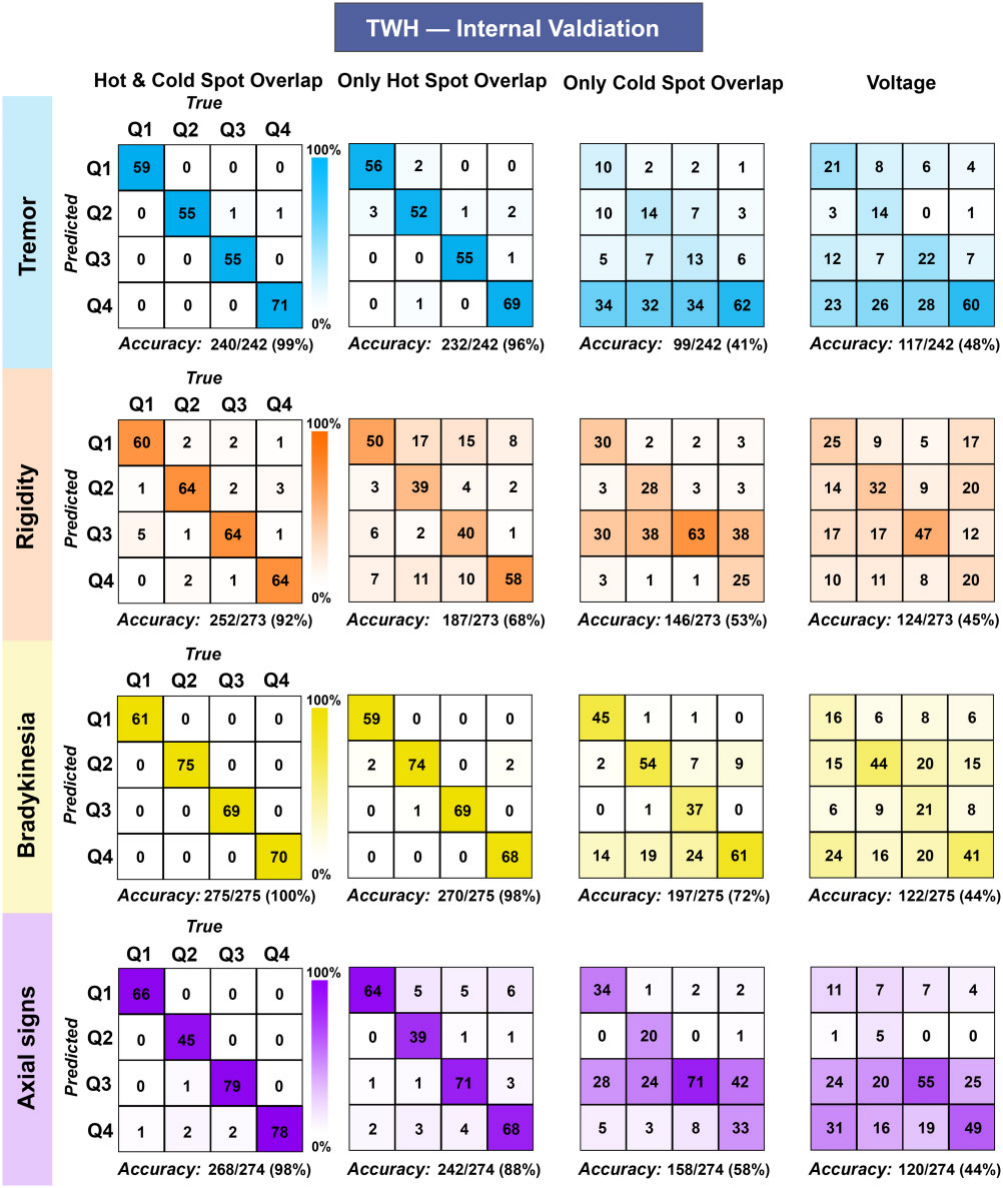
**Machine learning model classification accuracy using TWH (Internal Validation).** Sign-specific accuracy matrices (4 × 4) classifying patients into quartiles of clinical changes (Q) obtained with SVM model (machine learning model). To ascertain the robustness of our model, we tested its accuracy with different inputs (VTA overlap with hot spots only and/or cold spots only). Voltage, a surrogate of VTA size, was also used as an input. The diagonal (top left—bottom right) represents patients correctly classified. Matrix columns and rows represent true and predicted data, respectively. TWH = Toronto Western Hospital; VTA = volume of tissue activated.

We validated the observed hot and cold spots using SVM, assessing whether the amount of VTA overlap with these areas could accurately explain sign-specific clinical outcomes (Supplementary Table 5). To ascertain the robustness of our model, we tested its accuracy with different inputs (overlap with hot spots only, cold spots only or both) (Fig. 3). The most accurate model was obtained when considering overlaps with both hot and cold spots. The model accuracy ranged from 92% to 100% when classifying the vast majority of patients into their quartiles of clinical changes for each sign (Fig. 3). Overlap with hot spots only did not perform as well as the combined model (accuracy 68–98%), but it outperformed the model using cold spots only (accuracy 41–72%), suggesting that hot spot location is a stronger determinant of clinical outcomes.

We also performed two additional SVM analyses to further interrogate our model. First, to control for the extent of stimulation, we also tested the model using voltage only as an input (rather than overlap with hot or cold spots). This model performed poorly (accuracy 44–48%, Fig. 3), highlighting that the location of stimulation (rather than the volume of stimulation) is the crucial determinant of treatment efficacy. Second, since baseline sign severity was significantly correlated with postoperative clinical change (Pearson's product-moment correlation, *P* < 0.001), we investigated whether removing the influence of (i.e. regressing out) the baseline sign score changed the accuracy of our model (Supplementary Fig. 2). When considering only the residuals (i.e. after removing baseline scores), the combined model efficacy only slightly decreased (accuracy 91–100%), highlighting the extent to which stimulation location, rather than disease severity, drives differences in clinical changes.

**Figure 4:**
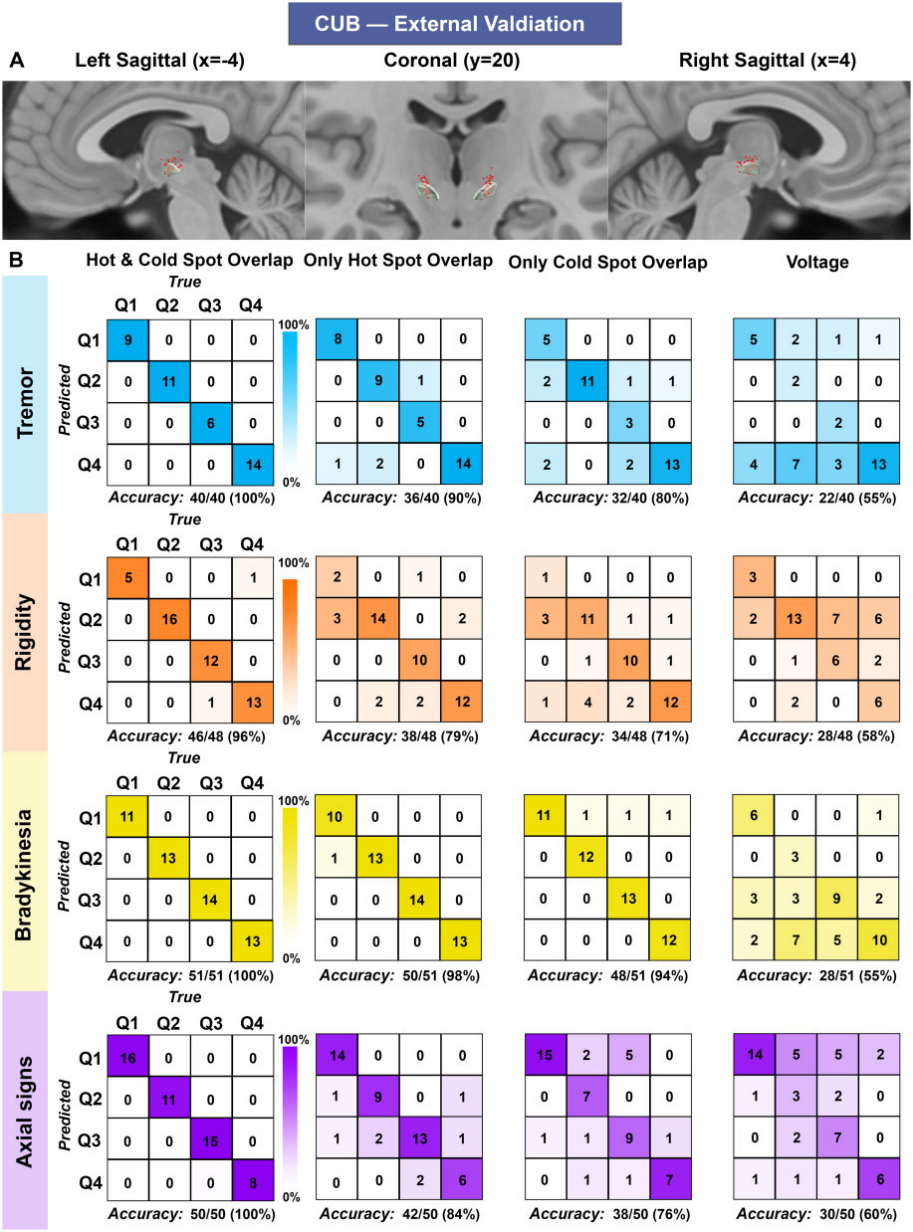
**Machine learning model classification accuracy using CUB (External Validation).** (**A**) CUB distribution of the active contacts (red dots) and the subthalamic nucleus (shaded green) are shown on sagittal (first and third column) and coronal (middle column) T_1_-weighted MRI (MNI ICBM 2009 b NLIN asymmetric). (**B**) Sign-specific accuracy matrices (4 × 4) classifying patients into quartiles of clinical changes (Q) obtained with SVM model (machine learning model). To ascertain the robustness of our model, we tested its accuracy with different inputs (VTA overlap with hot spots only and/or cold spots only). Voltage, a surrogate of VTA size, was also used as an input. The diagonal (top left—bottom right) represents patients correctly classified. Matrix columns and rows represent true and predicted data, respectively. CUB = Charité-Universitätsmedizin Berlin; VTA = volume of tissue activated.

Finally, we applied our SVM model to an unseen dataset from a second, independent institution (CUB). We tested whether overlap of these patients’ VTAs with sign-specific hot and cold spots derived from the TWH cohort also predicted clinical improvement (Supplementary Table 5). Again, the SVM model using both hot and cold spots overlap outperformed the other models, predicting the clinical improvement quartile with high accuracy (accuracy 96–100%) (Fig. 4).

### Structural connectivity patterns of hot spots

**Figure 5:**
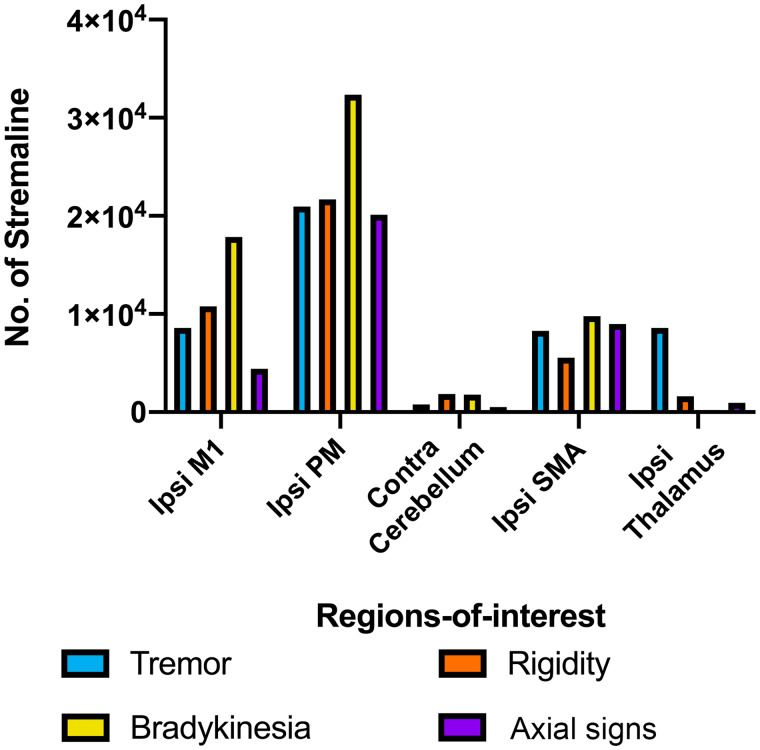
**Sign-specific hot spots structural connectivity.** Sign-specific hot spots were iteratively seeded (50 000 streamlines) into high-quality normative diffusion-weighted imaging^22^ to investigate patterns of connectivity to motor regions including primary motor cortex (M1), premotor cortex (PM), cerebellum, supplementary motor area and thalamus. Contra = contralateral; Ipsi = ipsilateral.

In an exploratory analysis, we seeded sign-specific hot spots in a high-quality normative diffusion-weighted imaging template to investigate whether motor regions previously implicated with motor improvement, demonstrated preferential structural connectivity to specific hot spots. Of the included motor regions-of-interest, the primary motor and premotor cortex received the most streamlines (Fig. 5). The thalamus was most connected with Hot_T_, whereas the primary motor and premotor cortex were most connected with Hot_B_.

### Analyses using different hot and cold spots

Additional hot and cold spots were computed using the complete UPDRS-III scores, which were primarily located in the STN with extension into the adjacent white matter, and in the thalamic ventral-intermediate nucleus area and adjacent white matter, respectively (Supplementary Fig. 3). When considering VTA overlap with both hot and cold spots, the model accuracy was 100%. Furthermore, additional hot and cold spots were computed using threshold cluster free enhancement. These spots were larger than the hot and cold spots obtained with the thresholding and binarizing method (Supplementary Fig. 4). When considering VTA overlap with both threshold cluster free enhancement hot and cold spots, the model accuracy was 100% for the four hot and cold spot labels.

## Discussion

Using a large cohort of Parkinson’s disease patients receiving STN-DBS, we defined brain areas associated with sign-specific optimal (i.e. hot spots) and suboptimal (i.e. cold spots) clinical efficacy. These areas were validated with in-sample and out-of-sample data using supervised machine learning. Based on the extent of stimulation overlap with these areas, our machine learning model was able to predict a patient’s clinical improvement in each of the four cardinal signs of Parkinson’s disease with high accuracy (≥92%). We also showed differences in structural connectivity between hot spots and motor regions-of-interest.

Similar to a recent study defining discriminative streamlines with respect to clinical response to DBS for obsessive-compulsive disorder,^23^ we used mass univariate analyses to define sign-specific hot and cold spots. This method identifies brain areas that discriminate optimal (i.e. positive *t*-values) and suboptimal (i.e. negative *t*-values) efficacy. Importantly, the absence of a brain area from our hot and cold spots labels does not mean that it has no contribution to clinical outcomes; rather, it means that, based on our data, this area was not sufficient to differentiate (i.e. low absolute *t*-values) between patients with optimal and suboptimal benefits. This distinction is important when comparing our results to those of previous studies, which often constructed probabilistic stimulation maps that reflect voxel-wise clinical outcomes.

Using probabilistic stimulation maps, a recent study^8^ defined and validated areas associated with improvement in rigidity and bradykinesia, which they found were largely located outside dorsolateral STN. In contrast to our findings, areas associated with tremor improvement were not significant in this earlier analysis. Another group^24^ also reported that the white matter adjacent to the dorsolateral STN played a crucial role for rigidity and bradykinesia improvement. We also found that a large proportion of Hot_R_ and Hot_T_ were located mostly adjacent to the STN, whereas Hot_B_ and Hot_A_ had larger overlap with the STN. A study^6^ demonstrated that tremor, rigidity and bradykinesia improved when the dorsolateral STN was stimulated. In line with subthalamotomy studies,^25^ these findings suggest that modulation of STN itself, as well as neighbouring structures, contribute to therapeutic benefits, highlighting the complex interactions with other circuits such as the hyperdirect pathway. Finally, studies using only active contacts (rather than VTAs) have reported that STN and adjacent structures, such as the zona incerta and substantia nigra, may contribute to bradykinesia and tremor improvement, respectively.^26^ Highlighting the importance of ‘sweet spots’ validation, our study and another group^8^ used machine learning and linear mixed-effect models, respectively. Rather than raising doubts about the utility of group-level neuroimaging DBS studies, these heterogenous findings should reflect the evolution of neuroimaging methods, with recent studies focusing on data validation.

We validated our hot and cold spots using machine learning—specifically SVM—predicting patients’ clinical outcomes with a high accuracy. Interestingly, although the position of active contact differed slightly between institutions, the external validation maintained a high accuracy, emphasizing the robustness of our model. In doing so, we validated the t-map thresholding and provided insights on the contribution of various inputs to model performance. Using VTA overlap with hot spots provided markedly higher accuracy than overlap with cold spots, suggesting that clinical improvement is better explained by the engagement of certain beneficial regions than the avoidance of unwanted ones. We also showed that stimulation location is the primary driver of clinical outcomes. Indeed, voltage—a surrogate of stimulation volume—was a poor predictor of improvement. Furthermore, after regressing out the relationship between preoperative sign severity and outcomes, the model’s predictive accuracy was largely maintained. These findings support the notion that therapeutic effects achieved with DBS hinge upon selective stimulation of specific areas.^3^

The magnitude of clinical improvement and the timepoint at which greatest improvement was achieved were different across Parkinson’s disease signs. This highlights the difficulty of programming in STN-DBS patients,^13^ which took at least 1.5 years before clinical outcomes were optimized. Interestingly, tremor—which responds with a variable time delay to DBS when compared to rigidity—took significantly longer to optimize than other signs.^14^ Programming is commonly driven by rigidity improvement, however, this may be associated with paradoxical deterioration of gait and akinesia. Our findings are in line with this established clinical notion, reporting higher efficacy for rigidity and tremor, relative to bradykinesia and axial signs.^13^^,^^27^ STN-DBS would benefit from novel lead designs and stimulation paradigms enabling concomitant improvement of different motor signs. Our data showed partial overlap of the rigidity hot spot with bradykinesia and axial signs hot spots, which may explain the concomitant improvement in rigidity and bradykinesia or axial signs in some patients. Knowledge of these hot spots and their spatial interactions with one another is important since quality of life in Parkinson’s disease patients is often driven by clinical signs other than rigidity such as bradykinesia.^28^

This study has several limitations. First, although our data included STN-DBS surgeries performed by three neurosurgeons over 20 years, our hot and cold spots were limited to the stimulated (i.e. sampled) areas. Second, since VTAs were flipped to a single side, we forfeited our ability to find possible side-specific results. Because the concept of a ‘dominant’ STN has been suggested,^29^ future studies should compare right- and left-sided findings without flipping, examining contralateral clinical changes to discern possible asymmetries in stimulation location responsible for clinical changes. Third, while the lead localization, image normalization and VTA modelling methods—as implemented in Lead-DBS—have been previously described, it is important to acknowledge that each step is associated with errors that diminish the precision with which stimulation volumes can be localized in the brain (see Supplementary material for more details). Fourth, while we retrospectively validated the hot and cold spots using an independent dataset from a second institution, we did not apply our findings prospectively. Finally, when defining hot and cold spots using the full statistical information (i.e. unbinarized, see Supplementary Fig. 4), their ability to predict clinical outcome was marginally improved, perhaps supporting the benefits of using the full statistical information in future machine learning applications.

The primary purpose of this study was not necessarily to investigate anatomical structures responsible for clinical benefits, but rather to demonstrate that VTA modelling combined with machine learning could identify and validate precise locations of stimulation responsible for Parkinson’s disease sign-specific clinical benefits. For the postoperative programming, hot spots—originally defined in standard (MNI) space—could be precisely identified in an individual patient following a personalized non-linear transformation. Similar to Devaluez et al.,^30^ we believe that guiding stimulation overlap with hot spots, which can be visualized with existing VTA software, could enable the preferential targeting of a patient’s most disabling Parkinson’s disease signs. For the treating physician, this is more easily performed with discrete and binarized hot spots, which as per our data can predict clinical efficacy with a high degree of accuracy. Novel electrode designs and stimulation paradigms allowing directional VTAs could theoretically shape the VTAs to preferentially overlap certain sign-specific hot spots, although this new technology remains to be validated. The next step after clinician choice of optimal VTA based on visual overlap would be a machine learning-driven software defining optimal VTA. Such a software would be able to handle non-binarized hot and cold spots, thereby enabling the use of the full statistical data and likely yielding superior accuracy. In sum, future studies could explore the utility of these areas as adjunct targeting and programming tools using clinician choice of optimal VTA based on visual overlap and, eventually, machine learning-driven VTA optimization.

In conclusion, we identified sign-specific hot and cold spots in STN-DBS and validated these areas using in-sample and out-of-sample datasets, showing that individual clinical outcomes are largely explained by the degree to which these discrete areas are stimulated. These areas could serve to personalize surgical planning and programming, allowing sign-specific *circuitopathies* to be targeted based on patients’ most disabling signs and maximizing quality of life improvement. Validating such areas is pertinent as novel DBS technologies, such as directional leads, enable more precise and flexible delivery of stimulation to desired areas. These findings also represent an important step in identifying the neuroanatomical elements responsible for the manifestation and alleviation of cardinal Parkinson’s disease signs.

## Supplementary material

Supplementary material is available at *Brain Communications* online.

## Supplementary Material

fcab027_Supplementary_Data

## Abbreviations

CUB =: Charité-Universitätsmedizin Berlin
DBS =: deep brain stimulation
STN =: subthalamic nucleus
SVM =: support vector machine
TWH =: Toronto Western Hospital
UPDRS =: Unified Parkinson's Disease Rating Scale
VTA =: volume of tissue activated
