# Supplementary material for: Sign-specific stimulation ‘hot’ and ‘cold’ spots in Parkinson’s disease validated with machine learning
Source: Brain Commun. 2021 Mar 10;3(2):fcab027. doi: 10.1093/braincomms/fcab027 (PMC8042250; doi:10.1093/braincomms/fcab027)
Supplement: fcab027_Supplementary_Data [file fcab027_supplementary_data.docx]

**Supplementary Material**

**Patient Population**

Surgeries were performed by one of three neurosurgeons (AML, MH, or SKK). Inclusion criteria were: i) presence of baseline and follow-up clinical data; ii) high spatial resolution pre-operative and immediate post-operative structural imaging; iii) bilateral STN electrode placement for PD. Patients were excluded if they had: i) pre-DBS surgical interventions (e.g., thalamotomy, pallidotomy); ii) confounding pre-DBS neurological co-morbidities other than the surgical indication (e.g., symptomatic stroke) or post-operative pathological intracranial processes, which prevented reliable clinical assessments.

**Adjustment for Disease Severity**

In addition to Med-OFF-DBS-ON, the corresponding pre-operative baseline (Med-ON) and post-operative UPDRS-III scores (Med-ON/DBS-ON) were collected for each patient to adjust for disease progression. The adjustment factor was determined by the difference between DBS-ON/Med-ON after and Med-ON prior to the surgery over the maximum score for a specific sign.

Adjustment factor: Sign_post – Sign_baseline * 100

Max_sign_score

Sign_post: sign-specific postoperative score (DBS-ON/Med-ON)

Sign_baseline: sign-specific baseline score (Med-ON)

Max_sign_score: sign maximum score

Only patients with a best postoperative score (Med-OFF/DBS-ON) >1 year after surgery and who showed worsening were adjusted for disease progression. The difference of DBS-ON/Med-ON was then multiplied by the adjustment factor. This adjustment was performed on a small fraction of patients and resulted in minor changes (Supplementary Table 2).

**Image Acquisition**

Patients underwent clinically required high spatial resolution structural imaging (i.e., T1-weighted three-dimensional spoiled gradient echo (3D-SPGR) at 3T (n=81, General Electric Signa HDxt scanner, voxel size 1×1×1 mm, TR 9.0 ms, TE 3.7 ms, flip angle 12°) or 1.5T (n=194, General Electric Signa Excite scanner, voxel size 1×1×1 mm, TR 12.4 ms, TE 5.3 ms, flip angle 20°) MRI for DBS surgery planning. To confirm electrode position, immediate postoperative 1.5T (n=272, similar acquisition parameters as pre-operative MRI) or CT (n=3, Toshiba Aquilion 64 V3.35 or Aquilion ONE V6.0; voxel size 0.5×0.5×3 mm; convolution kernel [filter] FC64) scans were acquired.

**Electrode Localization and VTA Estimation**

To perform group-level analysis, an established DBS-specific pipeline (Lead-DBS v2.0 software, https://www.lead-dbs.org/) was used to (1) localize and (2) normalize individual DBS electrodes to standard space followed by (3) volume of tissue activated (VTA) estimation.

Following an initial semi-automatic trajectory reconstruction, DBS electrodes were then manually localized by two experienced users (>1000 electrodes) until consensus (GJBE and AB) was reached. While conducting the localization, they were blinded to clinical outcomes. Then, immediate post-operative images were rigidly registered to the pre-operative MRI using SPM12 (https://www.fil.ion.ucl.ac.uk/spm/software/spm12/). Subsequently, the post-operative images were non-linearly normalized to MNI space (ICBM 2009b NLIN asymmetric) using “effective low variance” ANTS (ANTS; http://stnava.github.io/ANTs/), with an additional subcortical affine refine transform utilized as necessary to correct for post-operative brain shift. These normalization settings were used because they were shown to optimally normalize subcortical structures relevant to DBS.^1, 2^ Finally, the rigid and non-linear transformations were used to warp the localized electrode into MNI space.

To approximate the spatial extent of the peri-electrode electric field wherein modulation of neuronal activity is assumed to occur, VTAs were computed as per  prior computational modelling approaches. First, a four-compartment volume conductor model (Iso2Mesh tool-box; http://iso2mesh.sourceforge.net/cgi-bin/index.cgi) was constructed. This model classified peri-electrode tissue as either gray matter (0.33 S/mm - nuclei were defined by the DISTAL atlas^3^), white matter (0.14 S/mm), electrode contacts (10^8^S/mm), or insulated electrode components (10^-16^ S/mm). The FieldTrip-SimBio finite element model (FEM) pipeline was then used to simulate the electric field distribution associated with the corresponding contact configuration and voltage settings (https://www.mrt.uni-jena.de/simbio/index.php/; http://fieldtriptoolbox.org). The gradient of this distribution was obtained with a derivation of the FEM solution and thresholded at 0.2 V/mm to define a binary VTA. As with other employed VTA modelling approaches,^4-6^ pulse width was not taken into consideration during this modelling. For each patient, DBS settings associated with the selected best post-operative scores were employed for modelling purposes.  Finally, to facilitate group-level analysis, left-sided VTAs were non-linearly flipped to the right using Lead-DBS.^7, 8^ Non-linear flip was used to ensure anatomical accuracy.

Although the Lead-DBS methodological approach has been used in numerous publications, we want to highlight its limitations. We acknowledge that the normalization between patient-specific and MNI space is a major source of error in terms of determining electrode position, and thus activation volume location. Lead-DBS normalization methods have been optimized for DBS neuroimaging analysis. They include i) multispectral warps using a high-resolution MNI template, ii) a specific subcortical refinement step, iii) brain shift correction, iv) use of a high-definition template, and v) phantom-validated electrode localization.^8, 9^ Results of the pipeline generate automated subthalamic nucleus and globus pallidus interna segmentations that are as precise as manual expert segmentations (when compared to inter-rater accuracies) on the structures in both low-resolution and high-quality datasets.^1^ This specific method was the top performer for subcortical image registrations in a recent comparative study that involved >10,000 nonlinear warps and a variety of normalization techniques. It has also been recently validated by another group.^2^ Since there are always unavoidable errors in moving from patient space to standard space, we have used an optimized approach, which allows us to conduct group-level analysis.

The estimation of the activation volumes, which are referred to as VTAs, has limitations. Specifically, the activation volume modelling used in this manuscript estimated the size and shape of electrical fields generated by DBS using an ‘E-field norm’ finite element method approach. This is not the most advanced or sophisticated model available. ^10, 11^ The volume conductor model used in our analysis is based on the relationships between activations of cable models and E-Field magnitudes published by a group in 2015.^4^ Similar to other publications,^4, 12^ we used an E-Field magnitude of >0.2 V/mm as a heuristic to estimate VTA volumes that best matches those derived from sophisticated and computationally intensive axon cable modelling.^11^ We acknowledge that this model has not yet been directly validated using animal models or electrophysiological recordings. However, recent comparative work has reaffirmed the general similarity of its results to the results of more sophisticated axon cable models, which involve computationally intensive simulations of axon membrane dynamics and ion channel kinetics.^10^

**Defining Sign-specific Sites of Optimal and Suboptimal Efficacy**

To define sign-specific discriminative sites of optimal (“hot spots”) and suboptimal (“cold spots”) efficacy, voxel-wise mass univariate analyses were performed using clinically weighted (by each sign cluster) VTAs. Each VTA was weighted using bilateral sign-specific change because i) while STN-DBS has been shown to primarily exert contralateral effects, it also has ipsilateral effects^13, 14^ and because ii) selected UPDRS-III items for each sign incorporated axial (i.e. non-lateralizing) items. Sign-specific voxel-wise group comparisons were then carried out using linear regression analysis; for each voxel, this approach compared the mean sign-specific clinical change associated with VTAs that overlapped the voxel in question with that of the non-overlapping VTAs. The resulting t-values discriminated voxels (i.e., brain areas) associated with optimal (positive t-values) and suboptimal (negative t-values) sign-specific outcomes when stimulated. Higher absolute t-values indicated that the voxel was highly discriminative for clinical efficacy. Mass univariate analysis is an established neuroimaging method and was recently employed to define discriminative white matter streamlines in the context of DBS for OCD.^15^

**Internal Validation of Sign-specific Sites of Optimal and Suboptimal Efficacy**

A supervised machine learning model, specifically support vector machine (SVM; Support-Vector machine multi-class classifier using e1071 package (version e1071_1.7-1) in R3.4.4) was used to validate the sign-specific hot and cold spots, including p-value threshold used. SVM is a linear machine learning model that creates a hyperplane that optimally separates and classifies a set of input data, maximizing the margin between the classes’ closest points. Once trained on input data, a given SVM model may be applied to classify other datasets.

**External Validation of Sign-specific Areas of Optimal and Suboptimal Efficacy**

As an external validation of the TWH hot and cold spots, we tested our SVM model on an unseen out-sample PD STN-DBS dataset from a separate institution (Charité – Universitätsmedizin Berlin (CUB)) (Fig. 1).^16^ Similar to the TWH cohort, the absolute difference between available post-operative (1-2 years after surgery) and baseline scores of the same UPDRS-III items were used to provide sign specific clinical improvement attributable to DBS. Patients with both baseline and best post-operative scores of 0 for a specific sign were again excluded from analysis. To facilitate group-level analysis, left-sided VTAs were also non-linearly flipped to the right using Lead-DBS.^7, 8^

As with the internal validation, the overlap of sign-specific CUB VTAs with their corresponding hot and cold spots (derived from TWH cohort) was used as SVM input. SVM output was the classification of CUB patients into sign-specific quartiles of predicted improvement. The slight differences into the quartile group size was due to patients having equal clinical score change. Once again, a separate SVM was trained to classify these patients into quartiles of improvement based on voltage alone.

**Analyses using different hot and cold spots**

For comparison with sign-specific sites of optimal and suboptimal efficacy, we also defined hot and cold spots for the complete UPDRS-III using the same methods. A mass univariate linear regression analysis was performed using best available post-operative complete UPDRS-III scores followed by thresholding (p_uncorrected_<0.01) and binarization of the resultant t-value map. Then, SVM classification was performed using the volume of overlap between individual VTAs and the hot and cold spots labels corresponding to complete UPDRS-III clinical change.

Additionally, to compare the prediction accuracy when using the complete statistical data, we also defined sign-specific hot and cold spots using threshold free cluster enhanced (TFCE) t-statistics rather than the mass univariate analysis with binarization (p_uncorrected_<0.01). This also allowed de-emphasizing brain areas with single voxels. Thus, additional sign-specific hot and cold spots were defined using threshold cluster free enhancement with clinically weighted (by each sign cluster) VTAs. This resulted in a map of cluster enhanced (TFCE) t-values discriminating voxels (i.e., brain areas) associated with optimal (positive t-values) and suboptimal (negative t-values) sign-specific outcomes when stimulated. These were thresholded at a TFCE t-value of 12 (p<0.0001 Bonferroni corrected for multiple comparisons). SVM classification was performed using the volume of overlap between individual VTAs and TFCE hot and cold spots labels corresponding to sign-specific clinical change.

**Structural Connectivity Patterns of Hot Spots**

The large averaged diffusion dataset of 1065 subjects from the human connectome project was constructed using multishell diffusion-weighted imaging. The scans were acquired with a multi-shell diffusion scheme, and the b-values were 990 ,1985 and 2980 s/mm2. The number of diffusion sampling directions were 90, 90, and 90, respectively, and the in-plane resolution and slice thickness were 1.25 mm. Diffusion data was reconstructed using q-space diffeomorphic reconstruction to obtain the spin distribution function.^17, 18^ A diffusion sampling length ratio of 1.7 was used. The output resolution was 1 mm isotropic. The restricted diffusion was quantified using restricted diffusion imaging.^19^

Then, within DSI-Studio (http://dsi-studio.labsolver.org/),^17^ sign-specific hot spots were used as seeds (50,000 streamlines) for deterministic tractography (River Kutta Integrator 4).^20^ The quantitative anisotropy threshold was randomly selected. The angular threshold was randomly selected from 15 degrees to 90 degrees. The step size was randomly selected from 0.5 voxel to 1.5 voxels. The fiber trajectories were smoothed by averaging the propagation direction with a percentage of the previous direction. The percentage was randomly selected from 0% to 95%. Tracks with length shorter than 30 or longer than 150 mm were discarded. A total of 50,000 seeds were placed.

Finally, structural connectivity between the sign-specific hot spots and relevant motor regions-of-interest (ROIs) was estimated. Specifically, the right (ipsilateral) primary motor cortex,^21^ supplementary motor area,^21^ premotor area (dorsal and ventral),^21^ thalamus,^3^ and left (contralateral) cerebellum^22^ were selected as ROIs based on previous studies.^16, 23, 24^

**Statistical Analyses**

Mean values and standard deviation were calculated. ANOVA and two-sample t-tests were used to compare demographics and interrogate for statistically significant differences using R (https://www.r-project.org/, version 3.4.4). Pearson's product-moment correlation was used to assess for relationships between baseline sign severity and post-operative clinical changes. A significance level of p<0.05 was used for these analyses. The voxel-wise linear regression analysis performed to identify discriminative areas of sign-specific improvement was performed in RMINC (https://github.com/Mouse-Imaging-Centre/RMINC).

**Supplementary Tables**

| **Patient Cohort/**  **Sign** | **Current Configuration (No. of VTA)** | **Voltage (volts)** | **Pulse Width (us)** | **Frequency (Hz, no. of patients)** |
| --- | --- | --- | --- | --- |
| **TWH** | | | | |
| **Tremor (n=242)** | Monopolar = 391  Bipolar = 55  Double mono. = 19  Interleaved = 19 | 3.2±1.0 | 61.2±9.8 | <100 = 63  100-150 = 268  >150 = 153 |
| **Rigidity (n=273)** | Monopolar = 446  Bipolar = 61  Double mono. = 21  Interleaved = 18 | 3.1±1.0 | 61.0±9.0 | <100 = 74  100-150 = 326  >150 = 145 |
| **Bradykinesia**  **(n=275)** | Monopolar = 460  Bipolar = 53  Double mono. = 15  Interleaved = 22 | 3.0±0.9 | 60.7±6.0 | <100 = 56  100-150 = 356  >150 = 549 |
| **Axial signs**  **(n=274)** | Monopolar = 453  Bipolar = 65  Double mono. = 12  Interleaved = 18 | 3.1±1.0 | 60.7±4.5 | <100 = 67  100-150 = 340  >150 = 141 |
| **CUB** | | | | |
| **Tremor**  **(n=40)** | Monopolar = 64  Bipolar = 1  Double mono. = 7  Interleaved = 8 | 2.4±0.8 | N/A* | N/A* |
| **Rigidity**  **(n=48)** | Monopolar = 74  Bipolar = 3  Double mono. = 8  Interleaved = 11 | 2.4±0.8 | N/A* | N/A* |
| **Bradykinesia**  **(n=51)** | Monopolar = 77  Bipolar = 3  Double mono. = 10  Interleaved = 12 | 2.4±0.8 | N/A* | N/A* |
| **Axial signs**  **(n=50)** | Monopolar = 76  Bipolar = 3  Double mono. = 10  Interleaved = 11 | 2.4±0.8 | N/A* | N/A* |

**Supplementary Table 1. DBS settings.** For the CUB cohort, * indicates that the precise pulse width and frequency were not available but the majority of the cohort were on 60 us and 130 Hz.

CUB = Charité – Universitätsmedizin Berlin; Hz = Hertz; N/A= Not available; TWH = Toronto Western Hospital; VTA = volume of tissue activated.

Note.—Unless otherwise stated the data are mean ± standard deviation

| **Sign** | **Laterality** | **Coordinates** | **TWH MNI Coordinates** | **CUB MNI Coordinates** | ***p*** |
| --- | --- | --- | --- | --- | --- |
| **Tremor** | Right | x | 12.1 ± 1.5 | 11.8 ± 0.9 | >0.05 |
|  |  | y | -13.1 ± 2.1 | -13.9 ± 2.1 | **0.012** |
|  |  | z | -4.5 ± 2.6 | -6.1 ± 1.8 | **<0.001** |
|  | Left | x | -12.2 ± 1.7 | -11.7 ± 4.1 | >0.05 |
|  |  | y | -12.9 ± 2.0 | -13.8 ± 2.1 | **0.01** |
|  |  | z | -4.5 ± 2.7 | -6.0 ± 2.2 | **<0.001** |
| **Rigidity** | Right | x | 12.1 ± 1.5 | 11.7 ± 1.0 | **0.027** |
|  |  | y | -13.1 ± 2.1 | -13.8 ± 2.0 | **0.035** |
|  |  | z | -4.6 ± 2.6 | -6.4 ± 1.8 | **<0.001** |
|  | Left | x | -12.2 ± 1.6 | -11.7 ± 3.7 | >0.05 |
|  |  | y | -13.0 ± 2.0 | -13.6 ± 2.0 | **0.030** |
|  |  | z | -4.4 ± 2.7 | -6.4 ± 2.0 | **<0.001** |
| **Bradykinesia** | Right | x | 12.1 ± 1.5 | 11.7 ± 1.0 | **0.027** |
|  |  | y | -13.1 ± 2.0 | -13.8 ± 2.0 | **0.012** |
|  |  | z | -4.5 ± 2.6 | -6.4 ± 1.9 | **<0.001** |
|  | Left | x | -12.2 ± 1.6 | -11.7 ± 3.6 | >0.05 |
|  |  | y | -13.0 ± 2.0 | -13.7 ± 2.0 | **0.018** |
|  |  | z | -4.4 ± 2.7 | -6.3 ± 2.1 | **<0.001** |
| **Axial signs** | Right | x | 12.1 ± 1.5 | 11.7 ± 1.0 | **0.026** |
|  |  | y | -13.1 ± 2.0 | -13.8 ± 1.9 | **0.038** |
|  |  | z | -4.5 ± 2.6 | -6.4 ± 1.8 | **<0.001** |
|  | Left | x | -12.2 ± 1.6 | -11.7 ± 3.6 | >0.05 |
|  |  | y | -12.9 ± 2.0 | -13.6 ± 2.0 | **0.023** |
|  |  | z | -4.3 ± 2.7 | -6.4 ± 2.0 | **<0.001** |

**Supplementary Table 2. Active contacts coordinates.** Active contacts (i.e., anodes) coordinates for TWH and CUB are displayed on the right and the left for each sign-specific cohort. For double monopolar and interleaved configurations, the active contact coordinates are the mean of both anodes. BOLD indicates statistical significance obtained using two-sample T-tests. MNI = Montreal Neurological Institute.

Note.—Unless otherwise specified, data are numbers of participants, ± standard deviation

| **Sign** | **No. of corrected patients’ scores** | **Unadjusted Clinical Change** | **Adjusted Clinical Change** | **P-Value** |
| --- | --- | --- | --- | --- |
| **Tremor (n=242)** | 15 | 4.9 ± 4.06 | 5.0 ± 4.2 | 0.714 |
| **Rigidity (n=273)** | 32 | 4.2 ± 3.55 | 4.3 ± 3.7 | 0.650 |
| **Bradykinesia**  **(n=275)** | 2 | 7.4 ± 6.10 | 7.4 ± 6.2 | 0.980 |
| **Axial signs**  **(n=274)** | 1 | 3.2 ± 2.72 | 3.2 ± 2.8 | 0.910 |

**Supplementary Table 3. Correction of sign-specific clinical outcomes.** Clinical change reflects the difference between the UPDRS-III at the time of follow-up and prior to surgery. Correction of the follow-up score was done using the UPDRS-III scores prior to surgery (medication-on) and at follow-up (medication-on/DBS-off) (see supplementary materials). P-value was obtained with a 2-sample 2-sided t-test comparing mean of unadjusted and adjusted changes. Unless otherwise stated the data are mean ± standard deviation. DBS = deep brain stimulation; UPDRS-III: motor section of the Unified Parkinson’s disease rating scale.

Note.—Unless otherwise stated the data are mean ± standard deviation.

| **Sign-specific hot spots** | **t-values** | **MNI Coordinates** | **Size (mm^3^)** | **Overlap with STN (%)** | **Overlap with sensorimotor STN (%)** | **Overlap with ZI (%)** |
| --- | --- | --- | --- | --- | --- | --- |
| Tremor | 4.22 | 12.5/-9/-5 | 151.5 | 8 | 0 | 12 |
| Rigidity | 3.56 | 13.5/-11/-10 | 65.9 | 9 | 4 | 4 |
| Bradykinesia | 5.51 | 13/-10/-7 | 154.1 | 36 | 17 | 6 |
| Axial signs | 3.93 | 12/-9/-5.5 | 50.3 | 38 | 10 | 0 |
| **Sign-specific cold spots** |  |  |  |  |  |  |
| Tremor | -2.85 | 9/-17.5/-4 | 8.9 | 0 | 0 | 4 |
| Rigidity | -3.25 | 12.5/-13.5/-0.5 | 24.4 | 0 | 0 | 0 |
| Bradykinesia | -4.30 | 12.5/-13/0.5 | 105.5 | 0 | 0 | 0 |
| Axial signs | -3.47 | 14.5/-14/-1 | 40.3 | 0 | 0 | 0 |

**Supplementary Table 4. Sign-specific hot and cold spots.** Maximum and minimum t-values (and their MNI coordinates) obtained from the mass univariate analysis are listed. Positive and negative t-values thresholded at uncorrected p<0.01 were used to define hot and cold spots, respectively. Sizes of the hot and cold spots as well as their overlap with the STN, sensorimotor STN, and ZI are shown.^3^ MNI = Montreal Neurological Institute; STN = subthalamic nucleus; ZI = zona incerta.

| **Patient Cohort/**  **Sign** | **VTA Overlap with hot spots (mm^3^)** | **VTA Overlap with cold spots (mm^3^)** | **Quartiles of Efficacy** |
| --- | --- | --- | --- |
| **TWH** | | | |
| **Tremor (n=242)** | 28.4±24.8 | 0.6±1.21 | Q1: [-2.8, 2.0)  Q2 : [2.0, 4.0)  Q3: [4.0, 7.0)  Q4: [7.0, 19.0) |
| **Rigidity (n=273)** | 5.2±7.1 | 2.6±4.3 | Q1: [-7.0, 2.0)  Q2: [2.0, 3.8)  Q3: [3.8, 6.65)  Q4: [6.65, 16) |
| **Bradykinesia**  **(n=275)** | 43.1±34.6 | 10.7±17.8 | Q1: [-9.0, 3.0)  Q2: [3.0, 7.0)  Q3: [7.0, 12.0)  Q4: [12.0, 26.2) |
| **Axial signs**  **(n=274)** | 11.2±11.1 | 3.0±5.7 | Q1: [-9.0, 1.5)  Q2: [1.5, 3.0)  Q3: [3.0, 5.0)  Q4: [5.0, 12.5) |
| **CUB** | | | |
| **Tremor**  **(n=40)** | 20.9±21.6 | 0.4±0.9 | Q1: [-4.0, 2.0)  Q2: [2.0, 4.5)  Q3: [4.5, 6.0)  Q4: [6.0, 15.0) |
| **Rigidity**  **(n=48)** | 8.6±9.2 | 0.4±.1.4 | Q1: [-1.0, 2.0)  Q2: [2.0, 4.0)  Q3: [4.0, 6.0)  Q4: [6.0, 13.0) |
| **Bradykinesia**  **(n=51)** | 52.2±4.3 | 2.1±6.1 | Q1: [-5.0, 3.0)  Q2: [3.0, 7.0)  Q3: [7.0, 11.5)  Q4: [11.5, 19.0) |
| **Axial signs**  **(n=50)** | 15.1±13.7 | 0.6±2.0 | Q1: [-9.0, 1.5)  Q2: [1.5, 3.0)  Q3: [3.0, 5.0)  Q4: [5.0, 12.5) |

**Supplementary Table 5. Volume of tissue activated overlap with sign-specific hot and cold spots.**

CUB = Charité – Universitätsmedizin Berlin; TWH = Toronto Western Hospital.

Note.—Unless otherwise stated the data are mean ± standard deviation.

**Supplementary Figures**


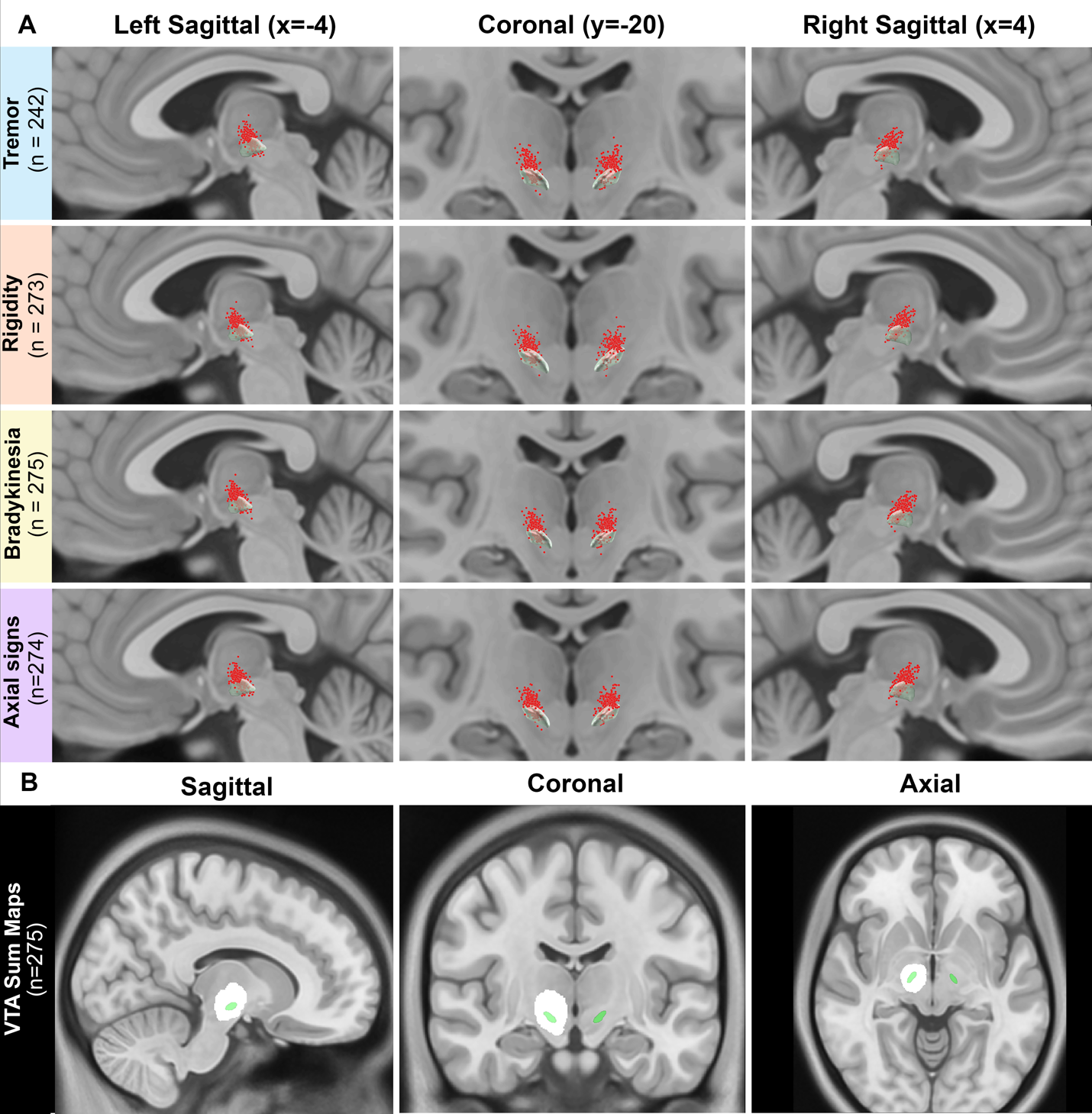


**Supplementary Figure 1: Active contact and area of stimulation at TWH.** (A) Distribution of the active contacts (red dots) and the subthalamic nucleus (shaded green) are shown. Mean MNI coordinates of the right and left active contacts are displayed. (B) Binarized sum maps of the volume of tissue activated of the TWH cohort (n=275) showing the spatial distribution of the stimulation. Data are projected on sagittal (first and third column) and coronal (middle column) T1-weighted MRI (MNI ICBM 2009b NLIN asymmetric). Mean active contact coordinates were also similar across the four signs (p>0.05, ANOVA). VTA = volume of tissue activated.


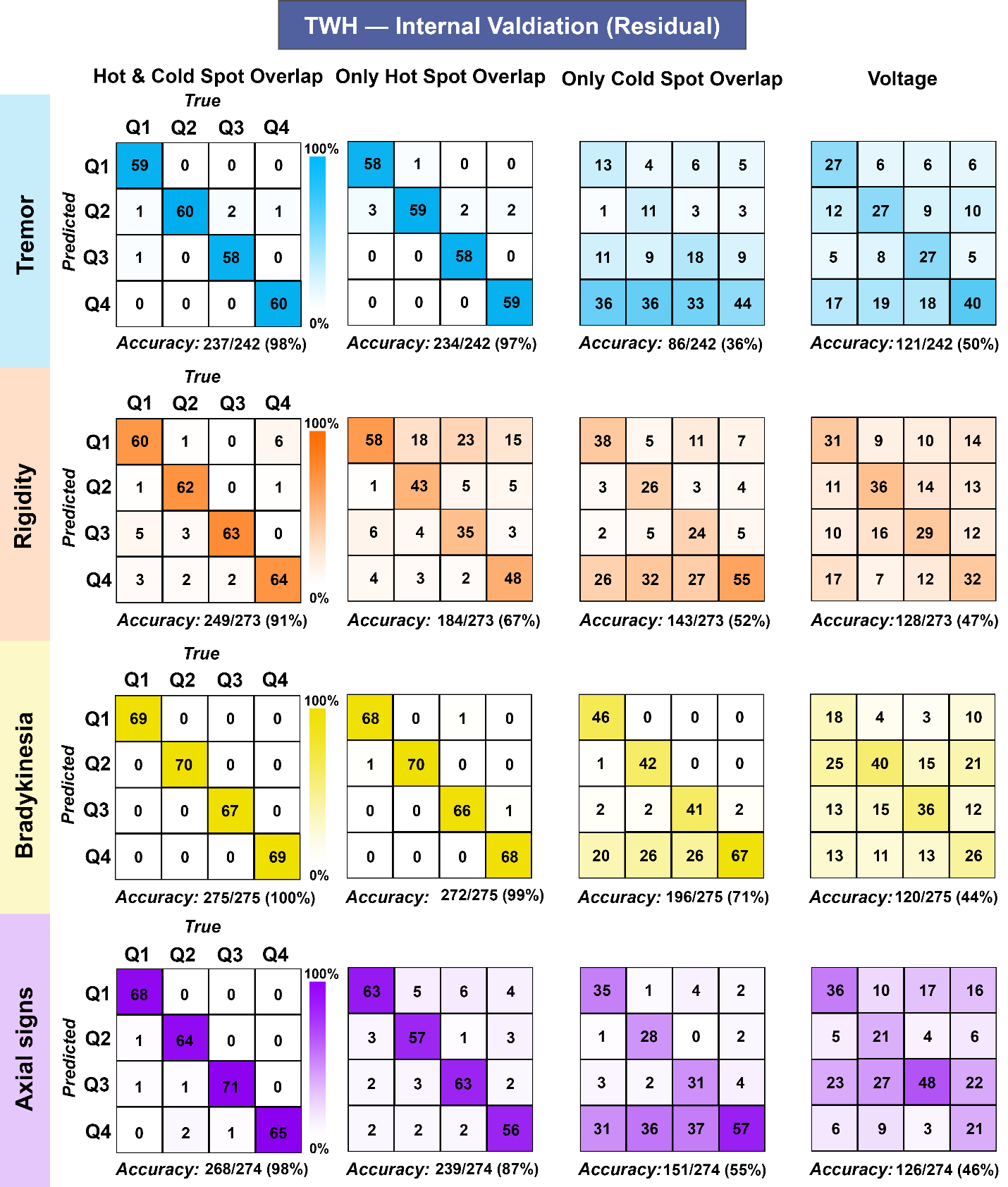


**Supplementary Figure 2: Machine learning model classification accuracy using TWH residuals (Internal Validation).** Since baseline sign severity was significantly correlated with postoperative clinical change (Pearson's product-moment correlation, p<0.001), we investigated whether removing the influence of the baseline sign score (i.e., regressing out) changed the accuracy of our model. Sign-specific accuracy matrices (4 x 4) classifying patients into quartiles of clinical changes (Q) obtained with support vector machine model (machine learning model). To ascertain the robustness of our model, we tested its accuracy with different inputs (VTA overlap with hot spots only and/or cold spots only). Voltage, a surrogate of VTA size, was also used as an input. The diagonal (top left – bottom right) represents patients correctly classified. Matrix columns and rows represent true and predicted data, respectively. TWH = Toronto Western Hospital; VTA = volume of tissue activated.


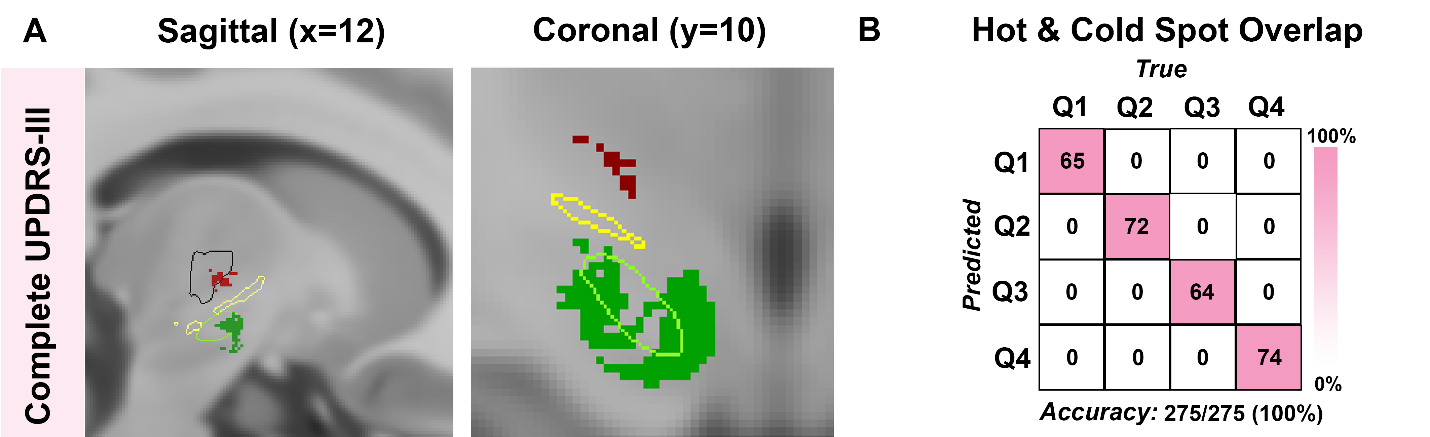


**Supplementary Figure 3: Area of clinical change when using complete UPDRS-III.** (A) Binarized areas of optimal (“hot spots”) and suboptimal (“cold spots”) efficacy were identified using mass univariate analysis (uncorrected p<0.01) in 275 PD-STN patients (mean preoperative UPDRS-III score = 36.5 ± 11.1 , mean adjusted clinical change = 19.7 ± 11.6). Hot (positive t-values, green) and cold (negative t-values, red) spots are shown. The subthalamic nucleus (green outline), zona incerta (yellow outline), and thalamic ventral intermediate nucleus (black outline) are projected on sagittal (left image) and coronal (middle image) T1-weighted MRI (MNI ICBM 2009b NLIN asymmetric). The nuclei outline were derived from nuclei labels^3^ using FSLeyes for visualization. (B) Machine learning model accuracy matrices (4 x 4) classifying patients into quartiles of clinical changes (Q) obtained with support vector machine model. PD = Parkinson’s disease; STN = subthalamic nucleus; UPDRS-III: motor section of the Unified Parkinson’s disease rating scale.


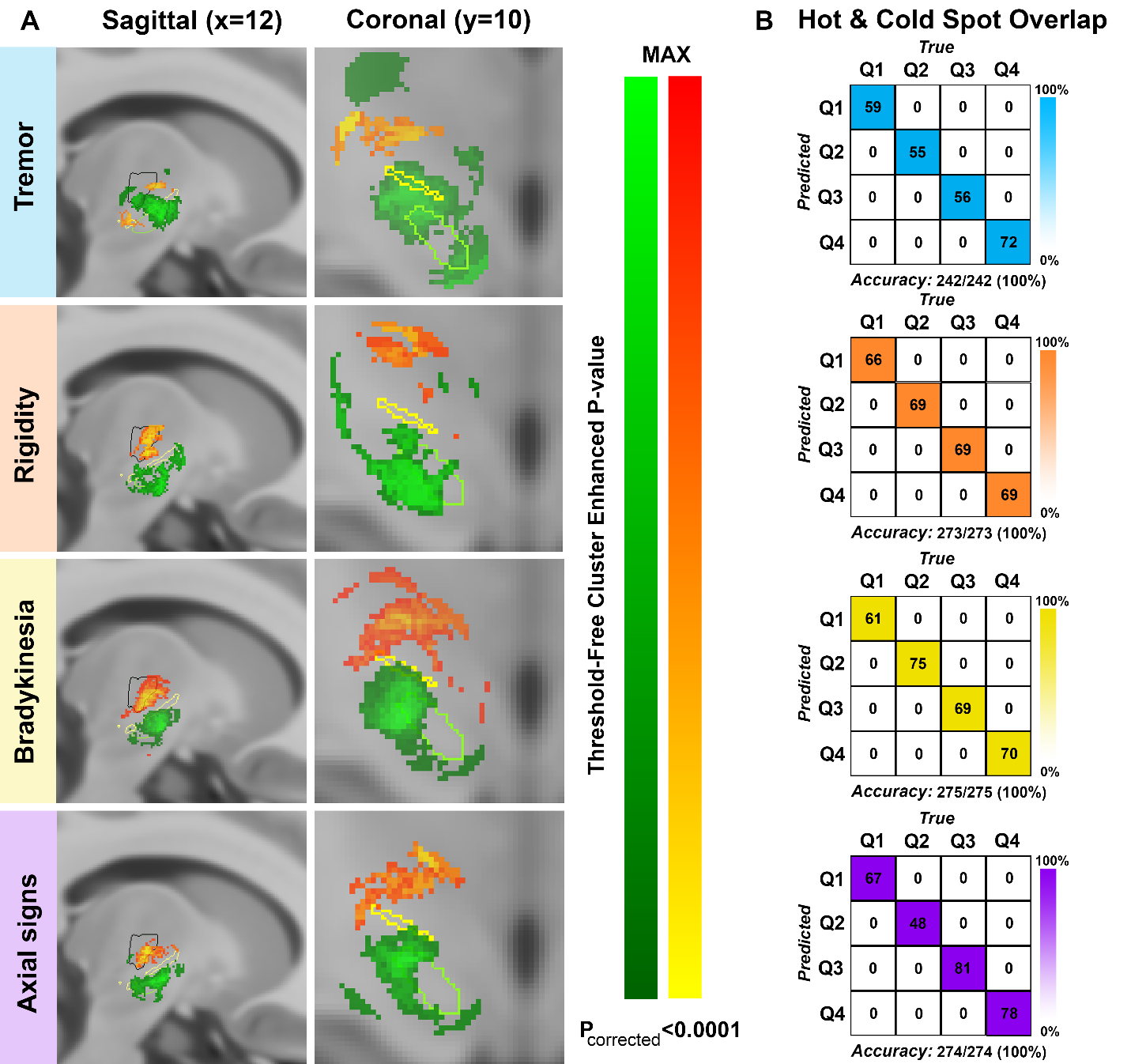


**Supplementary Figure 4: Sign-specific area of clinical change when using a different hot and cold spot method.** (A) Sign-specific hot and cold spots were defined using threshold free cluster enhanced (TFCE) t-statistics rather than the mass univariate analysis with binarization (p_uncorrected_<0.01). This resulted in a map of cluster enhanced (TFCE) t-values discriminating voxels (i.e., brain areas) associated with optimal (positive t-values) and suboptimal (negative t-values) sign-specific outcomes when stimulated. These were thresholded at a TFCE t-value of 12 (p<0.0001 Bonferroni corrected for multiple comparisons). Hot (positive cluster enhanced TFCE t-values, green) and cold (negative cluster enhanced TFCE t-values, red) spots are shown. The subthalamic nucleus (green outline), zona incerta (yellow outline), and thalamic ventral intermediate nucleus (black outline) are projected on sagittal (left image) and coronal (middle image) T1-weighted MRI (MNI ICBM 2009b NLIN asymmetric). The nuclei outline were derived from nuclei labels^3^ using FSLeyes for visualization. (B) Machine learning model accuracy matrices (4 x 4) classifying patients into quartiles of clinical changes (Q) obtained with support vector machine model.

**References**

1. Ewert S, Horn A, Finkel F, Li N, Kuhn AA, Herrington TM. Optimization and comparative evaluation of nonlinear deformation algorithms for atlas-based segmentation of DBS target nuclei. *Neuroimage*. Jan 1 2019;184:586-598. doi:10.1016/j.neuroimage.2018.09.061

2. Vogel D, Shah A, Coste J, Lemaire JJ, Wardell K, Hemm S. Anatomical brain structures normalization for deep brain stimulation in movement disorders. *Neuroimage Clin*. Apr 25 2020;27:102271. doi:10.1016/j.nicl.2020.102271

3. Ewert S, Plettig P, Li N, et al. Toward defining deep brain stimulation targets in MNI space: A subcortical atlas based on multimodal MRI, histology and structural connectivity. *Neuroimage*. Apr 15 2018;170:271-282. doi:10.1016/j.neuroimage.2017.05.015

4. Astrom M, Diczfalusy E, Martens H, Wardell K. Relationship between neural activation and electric field distribution during deep brain stimulation. *IEEE Trans Biomed Eng*. Feb 2015;62(2):664-672. doi:10.1109/TBME.2014.2363494

5. Chaturvedi A, Lujan JL, McIntyre CC. Artificial neural network based characterization of the volume of tissue activated during deep brain stimulation. *J Neural Eng*. Oct 2013;10(5):056023. doi:10.1088/1741-2560/10/5/056023

6. D'Haese PF, Pallavaram S, Li R, et al. CranialVault and its CRAVE tools: a clinical computer assistance system for deep brain stimulation (DBS) therapy. *Med Image Anal*. Apr 2012;16(3):744-53. doi:10.1016/j.media.2010.07.009

7. Dembek TA, Roediger J, Horn A, et al. Probabilistic sweet spots predict motor outcome for deep brain stimulation in Parkinson disease. *Ann Neurol*. Oct 2019;86(4):527-538. doi:10.1002/ana.25567

8. Horn A, Li N, Dembek TA, et al. Lead-DBS v2: Towards a comprehensive pipeline for deep brain stimulation imaging. *Neuroimage*. Jan 1 2019;184:293-316. doi:10.1016/j.neuroimage.2018.08.068

9. Husch A, M VP, Gemmar P, Goncalves J, Hertel F. PaCER - A fully automated method for electrode trajectory and contact reconstruction in deep brain stimulation. *Neuroimage Clin*. 2018;17:80-89. doi:10.1016/j.nicl.2017.10.004

10. Duffley G, Anderson DN, Vorwerk J, Dorval AD, Butson CR. Evaluation of methodologies for computing the deep brain stimulation volume of tissue activated. *J Neural Eng*. Oct 29 2019;16(6):066024. doi:10.1088/1741-2552/ab3c95

11. Gunalan K, Howell B, McIntyre CC. Quantifying axonal responses in patient-specific models of subthalamic deep brain stimulation. *Neuroimage*. May 15 2018;172:263-277. doi:10.1016/j.neuroimage.2018.01.015

12. Vasques X, Cif L, Hess O, Gavarini S, Mennessier G, Coubes P. Stereotactic model of the electrical distribution within the internal globus pallidus during deep brain stimulation. *J Comput Neurosci*. Feb 2009;26(1):109-18. doi:10.1007/s10827-008-0101-y

13. Shemisa K, Hass CJ, Foote KD, et al. Unilateral deep brain stimulation surgery in Parkinson's disease improves ipsilateral symptoms regardless of laterality. *Parkinsonism Relat Disord*. Dec 2011;17(10):745-8. doi:10.1016/j.parkreldis.2011.07.010

14. Toleikis JR, Metman LV, Pilitsis JG, Barborica A, Toleikis SC, Bakay RA. Effect of intraoperative subthalamic nucleus DBS on human single-unit activity in the ipsilateral and contralateral subthalamic nucleus. *J Neurosurg*. May 2012;116(5):1134-43. doi:10.3171/2011.12.JNS102176

15. Li N, Baldermann JC, Kibleur A, et al. A unified connectomic target for deep brain stimulation in obsessive-compulsive disorder. *Nat Commun*. Jul 3 2020;11(1):3364. doi:10.1038/s41467-020-16734-3

16. Horn A, Reich M, Vorwerk J, et al. Connectivity Predicts deep brain stimulation outcome in Parkinson disease. *Ann Neurol*. Jul 2017;82(1):67-78. doi:10.1002/ana.24974

17. Yeh FC, Tseng WY. NTU-90: a high angular resolution brain atlas constructed by q-space diffeomorphic reconstruction. *Neuroimage*. Sep 1 2011;58(1):91-9. doi:10.1016/j.neuroimage.2011.06.021

18. Yeh FC, Wedeen VJ, Tseng WY. Generalized q-sampling imaging. *IEEE Trans Med Imaging*. Sep 2010;29(9):1626-35. doi:10.1109/TMI.2010.2045126

19. Yeh FC, Liu L, Hitchens TK, Wu YL. Mapping immune cell infiltration using restricted diffusion MRI. *Magn Reson Med*. Feb 2017;77(2):603-612. doi:10.1002/mrm.26143

20. Yeh FC, Verstynen TD, Wang Y, Fernandez-Miranda JC, Tseng WY. Deterministic diffusion fiber tracking improved by quantitative anisotropy. *PLoS One*. 2013;8(11):e80713. doi:10.1371/journal.pone.0080713

21. Mayka MA, Corcos DM, Leurgans SE, Vaillancourt DE. Three-dimensional locations and boundaries of motor and premotor cortices as defined by functional brain imaging: a meta-analysis. *Neuroimage*. Jul 15 2006;31(4):1453-74. doi:10.1016/j.neuroimage.2006.02.004

22. Diedrichsen J. A spatially unbiased atlas template of the human cerebellum. *Neuroimage*. Oct 15 2006;33(1):127-38. doi:10.1016/j.neuroimage.2006.05.056

23. Akram H, Sotiropoulos SN, Jbabdi S, et al. Subthalamic deep brain stimulation sweet spots and hyperdirect cortical connectivity in Parkinson's disease. *Neuroimage*. Sep 2017;158:332-345. doi:10.1016/j.neuroimage.2017.07.012

24. Al-Fatly B, Ewert S, Kubler D, Kroneberg D, Horn A, Kuhn AA. Connectivity profile of thalamic deep brain stimulation to effectively treat essential tremor. *Brain*. Oct 1 2019;142(10):3086-3098. doi:10.1093/brain/awz236
